# Novel elbow-forearm support orthosis for post-stroke glenohumeral subluxation: a randomized controlled trial protocol

**DOI:** 10.3389/fneur.2026.1812255

**Published:** 2026-03-20

**Authors:** Hanbo Chen, Yong Lou, Weifeng Wen, Yongliang Guo, Zeyue Wen, Si Chen, Shujuan Huang, Xiao Lv

**Affiliations:** 1 Department of Rehabilitation Therapy, Guangdong Sanjiu Brain Hospital, Guangzhou, China; 2 Department of Rehabilitation Medicine, Guangdong Sanjiu Brain Hospital, Guangzhou, China

**Keywords:** elbow-forearm support, glenohumeral subluxation, orthosis, post-stroke, protocol

## Abstract

**Background:**

Glenohumeral subluxation (GHS) is a common complication following stroke, with an incidence rate of up to 73% in the acute phase, significantly impairing upper limb functional recovery and activities of daily living. Conventional support devices, such as the Harris hemiplegic sling and Bobath sling, have limitations including tendency to slip and displacement, resulting in incomplete reduction. To address these challenges, our research team has developed a novel 3D-printed elbow-forearm support orthotic device.

**Methods:**

This study employs a single-center, prospective, assessor-blinded randomized controlled trial design. We plan to recruit 60 post-stroke patients with GHS, who will be randomly allocated to either the intervention group (elbow-forearm support orthosis) or the control group (conventional shoulder sling). The intervention period is 4 weeks with 6 h of daily wear, followed by an 8-week follow-up period (total observation period of 12 weeks). The primary outcome measures are vertical distance (VD) and horizontal distance (HD) from the inferior margin of the acromion to the center of the humeral head. Secondary outcome measures include the Modified Ashworth Scale, Visual Analog Scale for pain, Fugl-Meyer Assessment for upper extremity motor function, and Modified Barthel Index. Statistical analysis will be performed using mixed-effects linear models.

**Discussion:**

The elbow-forearm support orthosis improves GHS through mechanisms including biomechanical support and neuromuscular modulation. This study employs standardized radiographic measurements, multidimensional assessment systems, and rigorous randomization design, which may provide high-quality evidence-based medicine for the treatment of post-stroke GHS. Study limitations include single-center design and limited sample size, necessitating multicenter large-scale studies for validation.

**Clinical trial registration:**

https://www.chictr.org.cn/, identifier ChiCTR1800018730.

## Introduction

Stroke is one of the leading causes of adult disability worldwide, with approximately 13.7 million new cases annually (1). Glenohumeral subluxation (GHS), a common complication following stroke, occurs in up to 73% of patients during the acute phase, with symptoms deteriorating over time in 67% of affected individuals (2). GHS not only reduces proprioception in the affected limb and exacerbates shoulder pain but also significantly restricts shoulder range of motion, ultimately impairing upper limb functional recovery and activities of daily living (3).

Current clinical approaches for managing GHS include functional electrical stimulation (4), kinesiotaping (5), and shoulder orthoses (6). However, meta-analysis results indicate that these conventional therapeutic techniques demonstrate suboptimal efficacy in improving post-stroke GHS (7). Notably, the only intervention recommended by the American Stroke Rehabilitation Guidelines is maintaining comfortable upper limb positioning and using supportive devices, though this recommendation carries a low level of recommendation and evidence quality (Class IIa recommendation; Level C evidence) (8).

Conventional support devices, such as the Harris hemiplegic sling and Bobath sling, while providing some immediate benefits, have significant limitations: these devices tend to slip due to gravitational forces, are prone to displacement during movement, can only achieve vertical reduction without anterior–posterior repositioning, and demonstrate incomplete overall reduction effects (9, 10). To address these challenges, our team has developed an innovative elbow-forearm support orthosis using 3D printing technology (Chinese Patent No.: CN201810839207.6). Preliminary studies demonstrate that this device significantly outperforms traditional traction-type orthoses in terms of immediate reduction effectiveness and user satisfaction (11).

The elbow-forearm support orthosis is theorized to function through the following mechanistic pathway: (1) Structural alignment phase: The adjustable waist fixation belt and elbow joint positioning device maintain humeral head alignment relative to the acromion, reducing the vertical and horizontal displacement characteristic of GHS; (2) Neuromuscular optimization phase: By maintaining optimal alignment, the device reduces abnormal traction forces on soft tissues, decreasing nociceptive input and allowing normalization of muscle tone through reduced protective muscle guarding; (3) Functional recovery phase: With improved alignment and reduced pain-driven inhibition, the affected rotator cuff muscles are positioned to engage more effectively during rehabilitation, facilitating sensorimotor integration and motor learning. This three-stage model grounds our outcome measurement strategy, where vertical distance (VD)/horizontal distance (HD) serve as objective biomarkers of the mechanical intervention, while spasticity and pain measures reflect the neuromuscular consequences of improved alignment, and motor/functional measures capture the downstream clinical benefits.

As a specialized neurorehabilitation center, the Department of Rehabilitation Medicine at Guangdong Sanjiu Brain Hospital treats a large volume of stroke patients annually and has accumulated extensive experience in rehabilitation therapy and orthotic applications. Based on our preliminary research findings and clinical practice requirements, we plan to conduct a randomized controlled trial to systematically evaluate the therapeutic efficacy of the elbow-forearm support orthosis for GHS in stroke patients.

## Study design and methods

This study employs a single-center, prospective, assessor-blinded randomized controlled trial design and will be conducted at the Department of Rehabilitation Medicine, Guangdong Sanjiu Brain Hospital, with a study duration of 12 months. The study protocol has been registered with the Chinese Clinical Trial Registry (ChiCTR1800018730) and approved by the Ethics Committee of Guangdong Sanjiu Brain Hospital [Ethics Approval No. 2018 (02)].

**Figure 1:**
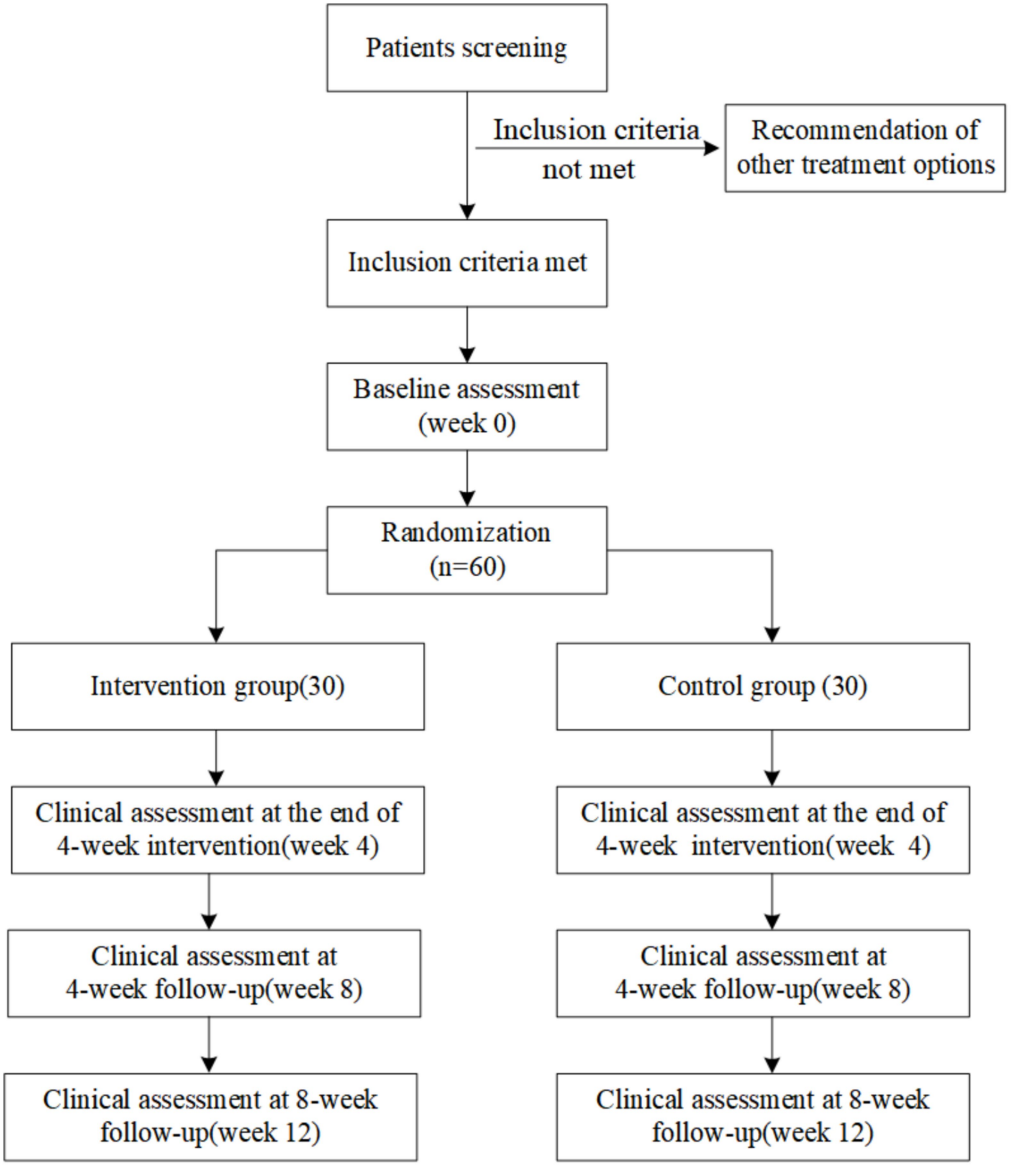
Study design (randomized controlled trials) and assessment time points (CONSORT chart).

The research study will adhere to the CONSORT (Consolidated Standards of Reporting Trials) Statement. The study protocol follows the SPIRIT (Standard Protocol Items: Recommendations for Interventional Trials) 2013 Statement (S1 Checklist). The flow diagram of study is shown in Figure 1. The schedule of enrollment, interventions, and assessments is described in Table 1.

**Table 1:** Schedule of enrolment, interventions, and assessments.

Item	Study period				
	Screening	Baseline	Treatment	Follow-up	
Timepoint	-1 week	0	4-weeks	8-weeks	12-weeks
Enrolment					
Eligibility screen	×				
Signed informed consent	×				
Randomization		×			
Interventions					
Intervention group		×	×		
Control group		×	×		
Assessments					
Primary outcome					
VD		×	×	×	×
HD		×	×	×	×
Secondary outcomes					
MAS		×	×	×	×
VAS		×	×	×	×
FMA-UE		×	×	×	×
MBI		×	×	×	×
Adverse Events		×	×	×	×

**×**Represents data collection timepoint. VD, Vertical distance; HD, Horizontal distance; MAS, Modified Ashworth Scale; VAS, Visual Analog Scale; FMA-UE, Fugl-Meyer Assessment of Upper Extremity function; MBI, Modified Barthel Index.

To ensure the scientific rigor and feasibility of randomization, this study employs computer-assisted stratified randomization using the Research Electronic Data Capture (REDCap) system. Stratification factors include age (≤50 years or >50 years) and time since stroke onset (≤30 days or >30 days). An independent biostatistician, who is not involved in patient enrollment, outcome assessment, or data analysis, will generate the randomization sequence using the minimization algorithm in SAS 9.4 software. The sequence will be encrypted and stored in a password-protected database accessible only to authorized personnel. The REDCap randomization system is independently managed by the hospital’s Information Technology Department, ensuring that research team members cannot access the randomization algorithm or modify the sequence. Group assignment information is automatically generated by the system upon completion of baseline assessment and baseline eligibility confirmation. Research coordinators responsible for patient enrollment, informed consent, and baseline assessments are blinded to future group allocations and do not have access to the randomization system until after baseline assessments are complete and all eligibility criteria are verified. Given the distinctive characteristics of the intervention devices, an assessor-blinded design will be implemented: all outcome measure assessments will be conducted by independent assessors who have received standardized training, do not participate in the treatment process, and remain blinded to patient group assignments. Additionally, patients will be explicitly instructed not to disclose their treatment group allocation during the assessment process. The effectiveness of allocation concealment will be assessed by comparing baseline characteristics between groups using chi-square test (categorical variables) and independent samples *t*-test (continuous variables). Significant imbalance in baseline characteristics would suggest inadequate allocation concealment.

This study comprises two parallel groups: the intervention group (elbow-forearm support orthosis) and the control group (conventional shoulder sling). During the 4-week intervention period, patients in both groups will use their respective devices regularly for 6 h daily while simultaneously receiving standard rehabilitation therapy according to current clinical guidelines. To ensure standardized intervention implementation, all healthcare personnel involved in treatment will receive systematic training in device usage and monitoring protocols. Following the completion of the 4-week intervention, an 8-week follow-up period will be conducted, during which patients will maintain daily rehabilitation diaries.

This study has established a rigorous quality control system to ensure data quality and trial integrity. All research procedures will be strictly executed according to standard operating procedures, with independent clinical research assistants conducting regular monitoring visits to verify protocol compliance and data accuracy. Trial data will be collected through electronic case report forms within the REDCap system, which incorporates built-in data range checks and validation rules to effectively ensure data quality. Additionally, a comprehensive adverse event recording and assessment mechanism has been established.

Outcome measure assessments will be conducted at baseline, and at 4, 8, and 12 weeks post-randomization. Primary outcome measures will employ standardized imaging techniques to measure VD and HD from the inferior margin of the acromion to the center of the humeral head. All imaging measurements will be performed by independent radiologists who are blinded to group allocation, ensuring objectivity and reliability of assessment results.

### Study participants

This study will recruit post-stroke patients with GHS from the Department of Rehabilitation Medicine at Guangdong Sanjiu Brain Hospital. All participants must meet the following criteria and provide informed consent.

#### Inclusion criteria

First-ever stroke (cerebral infarction or intracerebral hemorrhage confirmed by imaging); time from stroke onset to enrollment ≤6 months; age 18–80 years; GHS confirmed by imaging; Modified Ashworth Scale score ≤3; Mini-Mental State Examination (MMSE) score ≥21 with ability to understand and cooperate with treatment; stable vital signs with indications for rehabilitation therapy.

#### Exclusion criteria

Organic pathology around the shoulder joint (fracture, dislocation, arthritis, etc.); history of previous shoulder surgery or severe trauma; contraindications to rehabilitation therapy (severe cardiopulmonary dysfunction, coagulation disorders, etc.); severe cognitive impairment or psychiatric disorders; unsuitability for orthotic device wear (skin lesions, allergic history, etc.); receipt of treatments affecting muscle tone within the past 4 weeks (botulinum toxin injection, nerve block, etc.); participation in other clinical trials that may interfere with assessment.

The research team will conduct systematic screening of potential participants. Eligible candidates will receive a participant information sheet, and research personnel will provide detailed answers to any questions. The informed consent process will be completed after a minimum 24-h consideration period. All research personnel will undergo uniform training and strictly adhere to standardized screening and informed consent procedures.

### Sample size calculation

The sample size calculation for this study is based on the primary outcome measures of VD and HD from the inferior margin of the acromion to the center of the humeral head. According to Kumar et al. (12), the expected difference for VD is 5.2 ± 6.8 mm, and for HD is 4.8 ± 6.2 mm. Since the sample size calculation based on VD difference yields a larger result, we chose to base our sample size estimation on VD.

With a two-sided significance level of *α* = 0.05 and power (1-*β*) = 0.80, we employed the sample size calculation formula for independent samples t-test. Calculations were performed using PASS software (version 15.0, NCSS, LLC., Kaysville, Utah, United States) (13). Based on the systematic review by Nadler et al. (6), similar studies with 6–12 week follow-up periods report dropout rates of 15–25%. Considering that this is a single-center study with a 12-week follow-up period, we adopted a relatively conservative estimate and set the dropout rate at 20%.

Based on these parameters, the minimum required sample size is 25 participants per group. Accounting for a 20% dropout rate, we determined to recruit 30 participants per group, for a total sample size of 60 participants. The recruitment period is anticipated to last 6 months; if the planned sample size is not achieved, the recruitment period will be appropriately extended. We acknowledge that our sample size calculation, while based on the conservative.

endpoint-only t-test approach, will be analyzed using mixed-effects linear models that leverage repeated measures and within-subject correlations. This mismatch is intentional and conservative: mixed models typically provide greater statistical power than endpoint-only comparisons. Thus, our sample size of *N* = 60 represents a conservative estimate and ensures adequate power across analysis approaches.

### Interventions

Both groups will receive standardized basic rehabilitation therapy protocols. During inpatient rehabilitation, both groups will uniformly implement a standardized rehabilitation protocol consisting of: 40 min/day of motor therapy (progressing through shoulder range of motion, scapular stabilization, grip/upper limb function, and functional task training, with advancement upon achieving ≥80% movement accuracy and pain VAS ≤ 4); 30 min/day of occupational therapy (standardized upper limb ADL training progressing from dependent to supervised to independent); and 20 min/day of electrical stimulation (10 Hz low-frequency pulses, pulse width 200 μs, biphasic square waveform; applied to the supraspinatus and posterior deltoid, adjusted to visible muscle contraction without pain.). All rehabilitation treatments will be implemented by a team of therapists who have received uniform training.

**Figure 2:**
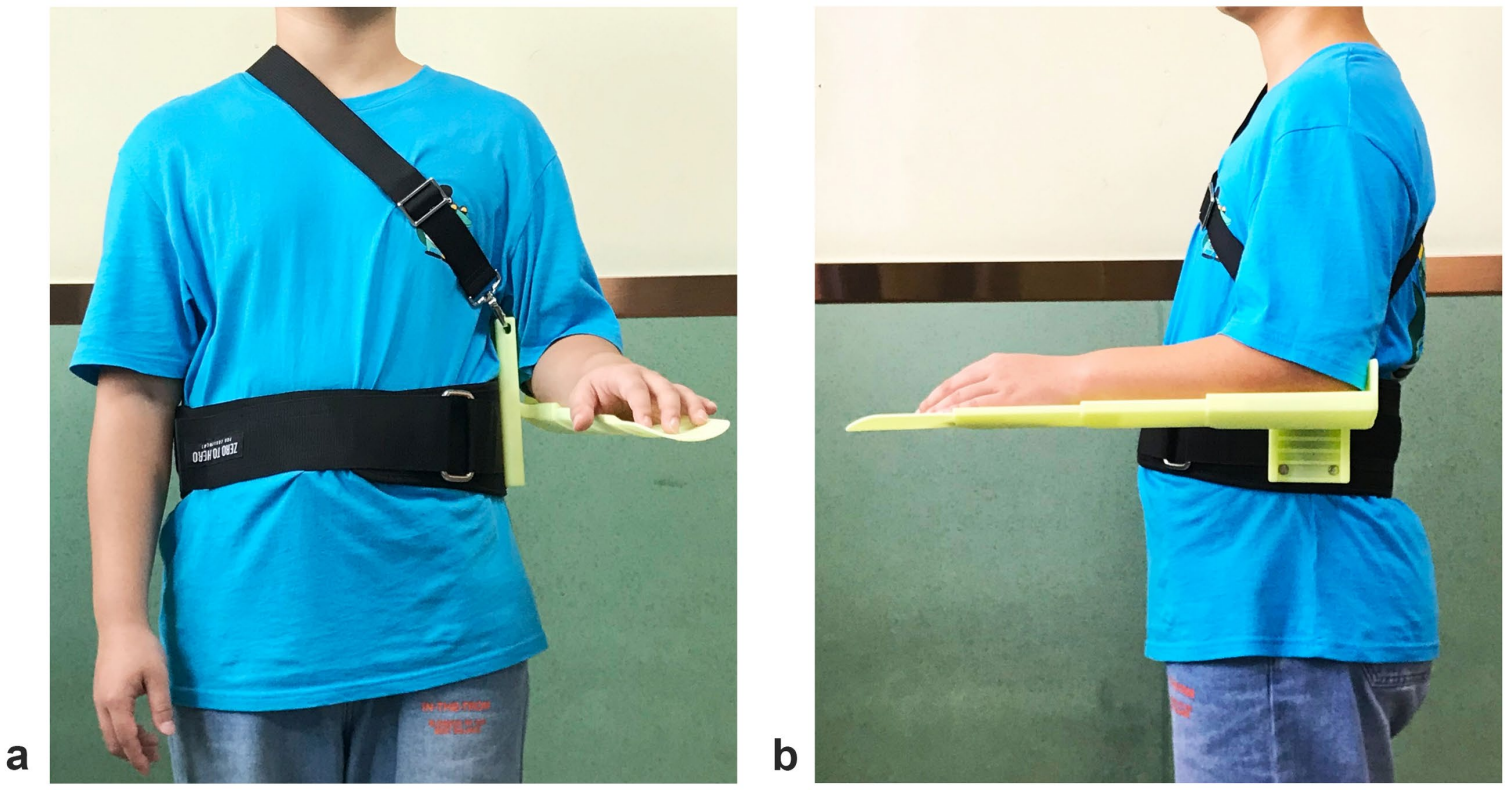
Effect of wearing the elbow forearm support orthosis (**a, b**: front view **b**. side view).

The intervention group will use a 3D-printed elbow-forearm support orthotic device developed by our research team in addition to routine care. The core structure of the device implements the alignment mechanism through three coordinated components: (1) the adjustable waist fixation belt (circumference adjustable from 60 to 120 cm) provides stable proximal fixation, counteracting gravitational downward translation of the humeral head; (2) the forearm support (length adjustable from 20 to 30 cm) provides lateral restraint at the forearm, limiting the inferior migration and anterior slippage common with conventional slings; (3) the elbow joint positioning device (angle adjustable from 0 to 90°) maintains the scapulohumeral relationship by preventing excessive scapular downward rotation and anterior tilting, which are biomechanical contributors to GHS. Together, these features maintain the humeral head centered beneath the acromion, the primary target of our primary outcome measures. To ensure reproducibility, a standardized fitting and adjustment protocol was established: (1) patient waist circumference and forearm length were measured and recorded prior to initial fitting; (2) belt tightness was deemed adequate when two fingers could be inserted beneath the belt without allowing easy slippage; (3) the forearm support length was adjusted to match the patient’s forearm length; and (4) palpation assessment of the humeral head position relative to the acromion was performed immediately after initial donning. The effect of wearing the elbow forearm brace is shown in Figure 2. The control group will use conventional traction-type traditional shoulder slings routinely used in clinical practice.

Both groups will begin device usage under the guidance of trained therapists, with daily wearing time of 6 h, distributed as 2 h each in the morning, afternoon, and evening, for a duration of 4 weeks. Therapists will perform weekly device inspections and adjustments according to a standardized protocol. Inspection items include skin integrity (presence of pressure marks, abrasions, or erythema), device positioning, belt tightness, forearm support alignment, elbow joint angle, and patient-reported comfort and pain levels (assessed using the Visual Analog Scale). Any deviation from the predefined criteria necessitates immediate adjustment and patient re-education.

During the study period, the use of other shoulder joint orthoses or fixation devices is strictly prohibited, as are invasive treatments such as botulinum toxin injections and intra-articular injections, as well as therapeutic modalities that may affect shoulder joint muscle tone, including transcranial magnetic stimulation and electrical stimulation. Additionally, patients are not permitted to participate in other clinical research projects.

### Outcome measures

The primary outcome measures of this study are the VD and HD from the inferior margin of the acromion to the center of the humeral head. These measurements will be obtained through standardized X-ray examinations. During examination, patients will adopt a standard sitting position with both upper limbs naturally hanging down and shoulders in neutral position. The X-ray tube center will be aligned at the height of the superior edge of the lateral end of the clavicle, horizontally positioned in line with the midline of the humeral head, angled 15° caudally, and positioned 1 meter from the patient. All imaging examinations will be performed by trained radiologic technicians, and image measurements will be independently completed by two senior radiologists blinded to group allocation. If the measurement difference between the two assessors exceeds 1 mm, a third senior radiologist will make the final determination.

Secondary outcome measures include: Modified Ashworth Scale (MAS) scores to assess the degree of spasticity in shoulder adductor and elbow flexor muscle groups (14); Visual Analog Scale (VAS) scores to evaluate shoulder pain intensity (15); Fugl-Meyer Assessment for Upper Extremity (FMA-UE) to assess upper limb motor function recovery (16); Modified Barthel Index (MBI) to evaluate activities of daily living, with particular attention to upper limb-related items (17).

Rationale for Outcome Measures Hierarchy: Our outcome selection reflects the theorized causal sequence of the intervention. Primary outcomes (VD/HD) directly quantify the device’s most immediate mechanical effect—humeral head repositioning. These measures are the most objective, least subject to confounding, and directly test the biomechanical hypothesis. Secondary outcomes are positioned at subsequent stages of the causal pathway: (a) MAS and VAS measure the neuromuscular benefits of improved alignment (reduced tone and pain); (b) FMA and MBI capture the functional consequences of improved motor control and reduced pain, which should emerge as downstream benefits. This staged framework ensures that outcome assessment timepoints are selected based on mechanistic expectations rather than statistical convenience.

### Predetermined visit schedule

All assessment measures will be obtained at baseline (0 weeks), 4 weeks of treatment, 8 weeks of treatment (4 weeks follow-up), and 12 weeks of treatment (8 weeks follow-up). Primary outcome X-ray examinations will be conducted in the hospital radiology department, while secondary outcome assessments will be completed at the rehabilitation medicine assessment center. Raw data from each assessment will be independently entered into electronic case report forms (REDCap system) by two research assistants and cross-verified. If patients are unable to attend scheduled assessments, home visits will be arranged. All follow-up data will be promptly recorded in standardized follow-up forms. The visit schedule for all assessments is presented in Table 1.

### Quality control, data collection, and safety measures

To ensure research quality, all therapists participating in the study must complete standardized training and pass assessments. To ensure protocol fidelity and detect potential performance bias: all therapists will complete standardized training covering rehabilitation content, progression algorithms, and technique consistency prior to study initiation; a detailed treatment log will be completed by therapists for each session, documenting specific exercises administered, number of repetitions, patient tolerance/pain level, and modifications; monthly fidelity audits will be conducted on a random 20% sample of treatment sessions, with blinded evaluation against standardized checklists; and treatment adherence rates will be calculated and reported by group to assess for differential treatment intensity. Patients will be required to maintain rehabilitation diaries recording daily device usage. During the home rehabilitation period, they will also record rehabilitation activity content, activity duration, and wearing time. The research team will assess patient compliance during each outcome evaluation.

This study employs the Research Electronic Data Capture (REDCap) cloud platform for data management. All data collection forms are structurally designed with built-in logic checking functions.

Potential adverse events in this study include: pressure-related skin injuries, local discomfort, and worsening shoulder pain. All adverse events will be reported in real-time through the REDCap system, and serious adverse events must be reported to the Ethics Committee within 24 h.

### Statistical analysis

This study will employ intention-to-treat (ITT) analysis as the primary analytical approach, with per-protocol (PP) analysis conducted as a sensitivity analysis. The ITT analysis set includes all randomized participants, while the PP analysis set includes participants who completed the full 12-week follow-up with device usage compliance ≥80%. All statistical analyses will be performed using R software version 4.2.0, with significance level set at *α* = 0.05 (two-sided test).

For baseline characteristics analysis, continuous variables will be presented as mean ± standard deviation or median (interquartile range) according to their distribution characteristics, and categorical variables will be presented as frequency (percentage). Between-group baseline comparisons will employ independent samples t-test or Mann–Whitney U test (for continuous variables), and chi-square test or Fisher’s exact test (for categorical variables).

Analysis of primary outcome measures (VD and HD) will employ mixed-effects linear models to account for the correlation of repeated measurements. The model will include fixed effects (treatment group, time point, and their interaction) and random effects (participant), with adjustment for baseline values and pre-specified covariates. Between-group differences will be expressed as least squares mean differences estimated from the model with their 95% confidence intervals.

Secondary outcome measures will be analyzed using different statistical methods. For ordinal categorical variables such as MAS scores and VAS scores, generalized estimating equations (GEE) models with cumulative logit link functions will be used. For continuous variables such as FMA-UE and MBI scores, the same mixed-effects model approach as the primary outcomes will be employed. Secondary outcome comparisons will be adjusted for multiple testing. For the primary analysis, we will apply Bonferroni correction to control family-wise.

error rate across secondary outcomes. However, given the expected correlation among secondary outcomes (pain, motor function, spasticity)—which may render Bonferroni overly conservative—we will additionally report results using the Holm-Bonferroni procedure (which maintains family-wise error control with less conservatism) and examine false discovery rate for comparative interpretation. Subgroup and sensitivity analyses are designated as exploratory given sample size limitations. Missing data will be handled using multiple imputation based on Markov Chain Monte Carlo (MCMC) methodology. The imputation model includes all baseline covariates (age, time since stroke, baseline GHS severity, stratification factors) and time-varying measurements of primary and secondary outcomes at available timepoints. We assume missing data are missing at random (MAR).

conditional on observed covariates—a reasonable assumption given that dropout is primarily due to logistical constraints (missed appointments) rather than outcome-dependent missingness. Twenty complete datasets will be generated, analyzed separately, and results combined using Rubin’s rules. Sensitivity analyses will assess robustness to departure from the MAR assumption.

This study will also conduct pre-specified subgroup analyses, including stratification by stroke type, baseline degree of GHS, and time from onset to enrollment. These analyses will assess heterogeneity of treatment effects by adding interaction terms between subgroup variables and treatment modality to the primary analysis models. Given sample size limitations, subgroup analysis results will be interpreted as exploratory analyses. To evaluate the robustness of results, a series of sensitivity analyses will be performed, including comparisons of different analysis sets, alternative missing data handling methods, and different statistical models.

The complete statistical analysis plan will be finalized and archived before enrollment of the first patient. The analytical process will be executed by an independent statistician who does not participate in study implementation and data collection.

### Ethics and dissemination

This study protocol has been approved by the Ethics Committee of Guangdong Sanjiu Brain Hospital [Ethics Approval No. 2018(02)], and will strictly adhere to the relevant provisions of the Declaration of Helsinki (2013 revision) and the International Ethical Guidelines for Biomedical Research Involving Human Subjects by the Council for International Organizations of Medical Sciences. Patients may withdraw from the study at any time without reason, and this will not affect their routine treatment. Study results will be submitted for publication in peer-reviewed journals within 12 months after completion of the final patient follow-up. Complete reporting will be provided regardless of whether results are positive or negative.

## Discussion

Post-stroke GHS is a common and challenging clinical problem that significantly impacts patients’ rehabilitation progress and quality of life (18, 19). Current research indicates that traditional shoulder slings have obvious limitations in preventing and treating GHS, including insufficient support for the humeral head, poor wearing comfort, and potential exacerbation of shoulder pain (9, 10, 20). Although some recent studies have attempted to improve orthotic design (21–23), these studies have mostly been observational studies or small-sample trials. Based on these issues, we designed this randomized controlled trial with the aim of systematically evaluating the effectiveness of a novel elbow-forearm support orthosis in treating post-stroke GHS through rigorous methodological design and standardized assessment systems.

This study has several significant methodological advantages. First, we employed standardized imaging measurement methods to assess the degree of GHS. By precisely measuring the vertical and horizontal distances from the inferior margin of the acromion to the center of the humeral head, treatment effects can be objectively quantified, avoiding the subjective bias of traditional clinical assessment methods (24). Second, this study established a comprehensive assessment system that includes not only objective imaging indicators but also incorporates clinically relevant indicators such as pain, spasticity, and functional status. This multidimensional assessment approach can more comprehensively reflect treatment effects (25). Third, we adopted a rigorous randomization allocation scheme with outcome assessments completed by independent evaluators, measures that can effectively control selection bias and measurement bias. Additionally, this study utilized the REDCap data management system, achieving electronic and standardized data collection, storage, and monitoring, significantly improving the efficiency and quality of data management. Radiation Safety Considerations: Primary outcome assessment involves radiographic imaging at baseline and weeks 4, 8, and 12. Each shoulder X-ray examination (standard AP view with 15° caudal angle) delivers an estimated absorbed dose of 0.01–0.02 mGy. The cumulative effective dose across the 4 examinations is approximately 0.04–0.08 mSv, which represents 2–3% of the annual background radiation exposure (~2–3 mSv) and remains well below ICRP occupational exposure limits (20 mSv/year). Estimated radiation-associated cancer risk is <0.001%. This level of radiation exposure is justified by the clinical necessity of objective radiographic assessment to rigorously evaluate orthotic efficacy. All participants provided informed consent acknowledging radiation exposure. All radiologic technicians are trained in ALARA (As Low As Reasonably Achievable) protocols to minimize unnecessary exposure. Finally, we developed detailed adverse event monitoring and management procedures, which are of great importance for evaluating the safety of interventions. These methodological advantages enable this study to provide higher-quality evidence-based medical evidence for the treatment of post-stroke GHS.

The mechanisms by which the elbow-forearm support orthosis improves post-stroke GHS may involve multiple aspects. From a biomechanical perspective, this device can effectively counteract the effects of gravity and abnormal muscle tone on the shoulder joint by providing upward support and inward thrust (26). Meanwhile, the device design also considers the biomechanical characteristics of the shoulder girdle, maintaining normal spatial relationships between the acromion and humeral head by limiting scapular downward rotation and anterior tilting (27).

From the perspective of neuromuscular regulation, the elbow-forearm support orthosis may function through multiple mechanisms. First, by providing continuous proprioceptive input, it promotes sensorimotor integration and helps improve proprioception of the affected upper limb (7). Second, the supportive action of the device reduces abnormal traction stimulation, potentially decreasing the degree of spasticity in shoulder muscle groups (28). Furthermore, by maintaining correct shoulder joint positioning, this device creates favorable conditions for functional recovery of the rotator cuff muscles, which is of great significance for preventing and improving GHS (24).

In summary, the elbow-forearm support orthosis may provide a comprehensive solution for treating post-stroke GHS through multiple mechanisms including biomechanical support, neuromuscular regulation, and functional adaptation.

This study also has some potential limitations. First, as a single-center study with a relatively limited sample size, this may affect the external validity of the research findings. Although we control for confounding factors through rigorous randomization and stratified design, the generalizability of the study results still requires further validation through multicenter large-sample studies. Second, considering the heterogeneity of stroke patients, the inclusion and exclusion criteria set in this study may limit the applicability of results to certain specific patient populations. For example, the exclusion of patients with severe cognitive dysfunction may affect the generalizability of study results in this population. Third, although this study employed standardized assessment methods, some functional assessment indicators may still be influenced by evaluator subjective judgment. These limitations suggest that we need to maintain a cautious attitude when interpreting study results, while also providing directions for improvement in future research.
